# Still teaching 1948: the unfinished decolonization of Nigerian medical education

**DOI:** 10.3389/fmed.2026.1914199

**Published:** 2026-10-02

**Authors:** Iveren Winifred Nyinoh, Ogo Agbor Ogo

**Affiliations:** 1 Department of Biosciences, Faculty of Health and Life Sciences, University of Exeter, Exeter, United Kingdom; 2 Department of Biochemistry, College of Health Sciences, Benue State University, Makurdi, Nigeria

**Keywords:** brain drain, curriculum reform, decolonization, epistemic justice, health professions education, indigenous knowledge, medical education, Nigeria

## Abstract

**Background:**

Despite growing global momentum to decolonize health professions education, Nigerian medical education remains underrepresented in mainstream decolonization scholarship.

**Approach:**

This is a narrative, interpretive perspective informed by selected Nigerian medical education literature and the authors' professional experience, rather than a systematic, scoping, or integrative review. It examines why the visibility gap exists, what it obscures, and traces the field's evolution from its colonial curriculum origin to current reform efforts.

**Key arguments:**

We argue that this limited visibility does not reflect an absence of reform, but a disconnect between locally driven educational innovations and terminology through which decolonization is recognized globally. Reforms are framed through alternative nomenclatures, such as community-based medical education, leaving them less legible. Nigerian medical education remains shaped by colonial legacies reflected in the 1948 University of London curriculum, Eurocentric biomedical dominance, Northern hegemony in knowledge production, publication inequalities, physician brain drain linked to neo-colonial dynamics of professional training, and chronic institutional underinvestment. At the same time, the Core Curriculum and Minimum Academic Standards signal a shifting landscape in curriculum reform.

**Implications:**

These challenges are structural and epistemological rather than merely curricular. Realizing emerging possibilities requires coordinated action from students, institutions, researchers, funders, regulators, and policy makers to move beyond symbolic diversification towards transformation that re-centers Nigerian and African perspectives in defining educational priorities. As a narrative perspective rather than a systematic evidence synthesis, the claims advanced here are best read as conceptual and hypothesis-generating and are not intended as an exhaustive account of the evidence base.

## 1 Introduction

Medical education shapes far more than clinical competence. It inculcates moral values, professional priorities, and epistemological foundations that define what counts as legitimate knowledge and whose experiences of health and illness are considered worth studying (1–3). It is therefore a site of considerable political consequence and one that requires continuous critical scrutiny. Internationally, there is increasing appreciation that health professions education must be fundamentally restructured to address the historic and structural inequities that colonial systems have embedded within curricula, institutions, and the very framework through which medicine understands itself (4, 5).

Efforts to decolonize health professions education have gained traction over the past decade. Decolonization, as Lokugamage et al. (6) argue, involves challenging and restructuring curricula shaped by colonial biases that systematically privilege white, Western, and male viewpoints while marginalizing other voices and forms of knowledge. More broadly, scholars emphasize that decolonization must fundamentally redress structural inequities and historical power imbalances rather than simply diversifying reading lists or adding marginalized perspectives as an afterthought (7, 8). Ndlovu-Gatsheni (9) has long argued that coloniality operates through epistemological as much as political and economic structures, a point relevant to medical education, where the boundaries of legitimate knowledge have historically been drawn in ways that exclude non-western healing traditions and local health epistemologies. These include indigenous herbal and spiritual healing systems practiced by Babalawo, Dibia, and similar traditional healers across Nigeria, and the knowledge held by traditional birth attendants who remain central to maternal healthcare delivery in many rural communities. Colonial administrators established institutions such as teaching hospitals, missionary-run medical services, and formal medical training systems based on European models of care. At the same time, indigenous healing traditions were often dismissed as backward, unscientific, or rooted in superstition, with western biomedicine positioned as the only legitimate and scientifically credible approach to healthcare (10). However, traditional medicine has long played an important role in the health and wellbeing of local communities. Empirical support for traditional medicine exists. Nyinoh et al. (11), for instance, documented the effective use of medicinal plants in traditional healing practices.

Within medical education, this complexity has generated a growing body of scholarship. Landmark student movements including #RhodesMustFall at the University of Cape Town (12) and #WhyIsMyCurriculumWhite at University College London (13) have pushed institutions to confront whose knowledge is valued and why. Despite this momentum, many institutions continue to struggle with translating decolonial aspirations into substantive curricular transformation, reflecting persistent uncertainty about the meaning, scope, and implementation of decolonizing curricula in practice (14, 15).

Nigeria stands out as a particularly compelling yet understudied case, given its status as Africa's most populated nation and home to the largest number of universities on the continent (16). The medical education system in Nigeria developed during the British colonial period with its first formal Faculty of Medicine established at University College Ibadan in 1948 (17, 18). Nigeria thus occupies a critical position in debates about decolonization in African higher education. Yet despite the growing global discourse, the Nigerian context has received limited systematic scholarly attention, with existing reviews concentrated in Global North settings or addressing African higher education in broad terms without the specificity needed to understand the Nigerian case (19, 20).

This paper argues that Nigerian medical education remains structurally and epistemologically colonized, that this has concrete consequences for patients, graduates, and health systems, and that meaningful transformation requires institutional reform, epistemological reorientation, and a fundamental redistribution of scholarly authority. These changes are both urgent and achievable.

### 1.1 Approach and scope

This paper is a non-systematic narrative and interpretive perspective, rather than a systematic, scoping, realist, or integrative review. It draws on a broad but purposively selected body of literature spanning Nigerian medical education, decolonization theory, curriculum reform, language and instruction, community-based training, and North-South global health partnerships. Sources were identified through iterative literature searching and citation tracking, prioritizing Nigerian and African scholarship where available, and were selected for direct relevance to the visibility gap and reform mechanisms discussed below, rather than through a predefined systematic search protocol. The analysis is substantially shaped by the authors' positionality and professional experience across Nigerian and UK medical education (Section 2), which informed both the questions asked and the interpretation of the literature. Because this is an interpretive rather than a systematic synthesis, the claims advanced in this paper should be read as conceptual argument and hypothesis-generation grounded in selected evidence, rather than as a comprehensive or representative account of the full evidence base; where direct Nigeria-specific outcome data are lacking, we say so explicitly.

## 2 Positionality

The authors approach this perspective from complementary positions within medical education in Nigeria and the United Kingdom. The first author has worked in Nigeria and transitioned to medical education in the UK, drawing on this experience alongside engagement with the relevant literature to examine Global North-South knowledge dynamics in medical education. The second author has worked within a Nigerian medical school for over a decade, providing sustained, ground-level insights into curricular, institutional, and epistemic structures. Together, the authors integrate experiential and scholarly perspectives to critically examine decolonizing medical education in Nigeria. We acknowledge that our positions do not encompass the perspectives of patients, indigenous knowledge holders, or students currently navigating the system, and we have sought to center their voices through the literature engaged with in this paper rather than substitute for them.

## 3 Findings

### 3.1 What is at stake: the implications of colonized medical education

The implications of colonized medical education plausibly extend well beyond the academy, although direct Nigeria-specific outcome evidence establishing these relationships remains limited. Contemporary healthcare education built on Eurocentric frameworks has been argued to contribute to gaps in diagnosis and mismatches between clinical practice and the cultural contexts of patients, with possible consequences for the quality and equity of care delivered to communities whose experiences are largely absent from medical training (21). A decolonized curriculum is argued, on largely conceptual grounds, to promote cultural humility, support critical and reflective thinking, and strengthen trust between healthcare providers and the communities they serve; these are plausible and important hypotheses that, to our knowledge, have not yet been directly tested in the Nigerian context.

In Nigeria, these consequences are acutely visible. With 2.9 physicians per 10,000 people, the ratio falls well short of the World Health Organization's recommended benchmark of 17 per 10,000 (22). Curricula heavily oriented towards Western biomedical models may inadequately prepare graduates for the epidemiological, social, and resource-related challenges of the Nigerian healthcare system, which may contribute to both poorer health outcomes and to accelerating physician emigration towards health systems for which their training is perceived to be better suited.

Findings from a 15-year cohort study revealed that close to 15% of Nigerian medical graduates leave the country within 15 years of completing their training, exacerbating already strained healthcare workforce shortages (23). Limited alignment between medical training and Nigeria's local health needs, communities, and epistemologies may reduce retention, making decolonization not only an equity concern but a health system sustainability imperative.

### 3.2 Structural and epistemological challenges: the enduring legacy of the 1948 colonial curriculum

The curriculum framework introduced at the University College Ibadan in 1948, modelled on the University of London syllabus under British colonial administration, oriented Nigerian medical education around Western disease profiles, biomedical epistemologies, and clinical priorities that reflected the health concerns of high-income countries rather than the epidemiological and social realities of Nigeria's population. This foundational template has never been sufficiently reformed. Contemporary Nigerian medical education remains structurally anchored in a colonial inheritance that continues to inform the content of curricula, the methods used to teach it, and which forms of knowledge are considered legitimate (24–26).

Training remains heavily centered on frameworks developed in and for high-income contexts, with limited formal integration of indigenous health knowledge, traditional healing practices, or locally relevant clinical approaches. Local health challenges including high burdens of infectious diseases, elevated maternal and neonatal mortality, and severe resource constraints require context-specific clinical reasoning that inherited Eurocentric curricula do not adequately cultivate (8, 27).

### 3.3 Northern hegemony and epistemic asymmetry

High-income country institutions exercise disproportionate authority over the frameworks, standards, and knowledge systems that define what counts as legitimate medical knowledge globally, a dynamic that Naidu (28) characterizes as a “Northern tilt” in which high-income-country-derived models function as universal standards imposed upon Global South contexts regardless of their relevance. Abimbola and Pai (8) argue that this asymmetry is structural: knowledge produced in the Global North is treated as universal and rigorous, while knowledge from the Global South is systematically positioned as local, particular, or insufficiently evidenced.

International accreditation standards, postgraduate fellowship systems, and publication hierarchies all implicitly favor Western frameworks, creating institutional incentives for Nigerian medical schools to orient their curricula towards models designed for high-income contexts. A study by Tamaki et al. (29) reveals an ongoing structural dependency in sub-Saharan African scholarship, in which international collaborations frequently exclude or marginalize local researchers from leadership roles, with funding, authorship, and agenda-setting authority concentrated in high-income country institutions. The consequence of this is a body of medical education evidence that systematically underrepresents African perspectives, priorities, and contexts even when research is ostensibly conducted on or about African populations.

### 3.4 Brain drain as neo-colonialism

The large-scale emigration of Nigerian-trained physicians to high-income countries is both a symptom and a perpetuator of colonial structural legacies in medical education. Ebeye and Lee (30) contend that by training African doctors to meet UK medical standards using resources financed by Nigerian taxpayers, the medical education system effectively channels graduates toward employment in Western healthcare systems, a contemporary form of neo-colonialism operating through professional training and accreditation rather than direct political control. Addressing brain drain is thus positioned not simply as a matter of retaining healthcare workers, but as an urgent decolonial imperative for health equity, one that demands accountability from high-income countries that benefit from recruiting globally, alongside structural reform of the educational systems that produce this misalignment (30, 31).

### 3.5 Language, institutional resistance, and resource constraints

English remains the exclusive medium of instruction in Nigerian medical schools, functioning as what Matahela (32) terms a “gate-keeping regime”, a structural mechanism that privileges students with strong English proficiency and creates barriers between graduates and the patients they serve. By contrast, European medical education demonstrates that global engagement need not require English-only instruction. Many countries continue to train medical students in national languages while using bilingual approaches that balance internationalization with local linguistic relevance, offering lessons for integrating indigenous languages (33). Studies in South-Western Nigeria on medical students are instructive, with 70.8% already using Yorùbá, an indigenous language, in clinical encounters despite receiving no formal training in doing so, and 73.7% support the formal integration of indigenous language training into the curriculum (34). We, however, recognize Nigeria's linguistic diversity, with over 300 spoken languages, and decolonizing medical education is unlikely to involve replacing English as the primary medium of instruction. Rather, it may require greater recognition and integration of clinically relevant indigenous regional languages (Hausa, Igbo, and Yorùbá) or Pidgin English within communication skills training, community placements, and patient-centered care. These findings reveal that indigenous language use is already a lived clinical reality, occurring informally and without curricular recognition.

Beyond the Nigerian-specific evidence on student language practice, a broader literature on foreign-language medical instruction clarifies the stakes of this issue. A systematic review by Hamad et al. (35) synthesizes evidence from countries that teach medicine in a language other than the students' first language, reporting consistent associations between foreign-language instruction and reduced comprehension, lower academic performance, diminished classroom participation, and weaker clinical communication, alongside effects on professional identity formation. Earlier work by Sabbour et al. (36) on Egyptian medical students and faculty members similarly documents persistent language barriers under English-medium instruction and divided attitudes towards Arabization of the curriculum, illustrating that the tension between international legibility and local linguistic accessibility is not unique to Nigeria. Qualitative work by Bouhani et al. (37) on breaking bad news among North African undergraduates further shows how instructional language and cultural framing shape sensitive, patient-facing clinical communication, reinforcing that language of instruction is not a peripheral logistical matter but a determinant of core clinical competence.

Taken together, this literature indicates that the case for indigenous-language integration should rest not on the assumption that it automatically improves communication or care, but on specific, evaluable outcomes. These include patient comprehension of clinical information, student confidence and participation in the clinical language actually used with patients, performance on structured communication assessments (for example, objective structured clinical examinations conducted partly in indigenous languages), the quality and appropriateness of interpreter use where full bilingual competence is not achievable, patient satisfaction with clinical encounters, and equity of access to language-integrated training across urban and rural training sites. Establishing these as explicit evaluation targets would allow indigenous-language integration to be assessed as an intervention with measurable effects rather than treated as a self-evidently beneficial reform.

Institutional conservatism and resource constraints compound these challenges. Medical education systems are resistant to fundamental change, with strong institutional investment in curricula and accreditation frameworks historically oriented towards Western models (26, 38). Chronic underfunding, brain drain of experienced academic staff, and the persistent influence of international accreditation standards that implicitly favor Western models all constrain the possibilities for meaningful reform. There is a risk that decolonization efforts become superficial, involving the addition of diverse content without addressing the underlying power structures and epistemological assumptions that shape the entire curriculum (14).

### 3.6 A comparative North African perspective

Nigeria is not alone in confronting the tension between an inherited foreign language of instruction and the linguistic realities of patient care. Egypt, Tunisia, and Morocco offer instructive comparators as medical education in each country has been shaped by colonial and post-colonial language politics that are distinct from, but structurally analogous to, the Nigerian case. In Egypt, English-medium instruction coexists with long-standing debate about Arabization of the medical curriculum; Sabbour et al. (36) found that both students and staff report persistent comprehension and communication difficulties under English-medium teaching, alongside divided opinion on whether Arabization would improve or compromise training quality and international legibility. In Tunisia and more broadly across the Maghreb, French retains a strong institutional role in medical education inherited from French colonial administration, producing a comparable gap between the language of instruction and the Arabic or Tamazight in which most patient care is actually conducted; Bouhani et al. (37) illustrate how this gap becomes especially consequential in emotionally sensitive clinical communication such as breaking bad news. Table 1 summarizes these parallels.

**Table 1:** Comparative language-of-instruction tensions in Nigeria and North Africa.

Country/Region	Inherited language of instruction	Locally dominant patient-facing language(s)	Nature of the reform debate
Nigeria	English	Hausa, Igbo, Yorùbá, Pidgin English	Informal student use of indigenous languages; calls for formal integration into clinical training Olajuyin et al. (34)
Egypt	English	Arabic	Ongoing Arabization debate; mixed student/staff attitudes Sabbour et al. (36)
Tunisia/Maghreb	French	Arabic, Tamazight	French retains institutional dominance; language gap acute in sensitive communication such as breaking bad news Bouhani et al. (37)

Across all three North African contexts and Nigeria, the underlying pattern is similar: an inherited colonial language retains institutional and international legitimacy, while the languages patients use remain informally, rather than formally, integrated into clinical training. This comparison suggests that Nigeria's language-integration agenda is part of a wider regional and postcolonial pattern, and that lessons on implementation and on evaluating whether Arabization- or indigenization-type reforms improve or complicate care, may usefully be drawn from these North African debates rather than developed in isolation.

## 4 Emerging possibilities

### 4.1 CCMAS as a potential institutional lever

Nigeria's recently revamped Benchmark Minimum Academic Standards (BMAS), now rephrased as the Core Curriculum and Minimum Academic Standards (CCMAS), was introduced in 2022. CCMAS represents the most concrete formal policy response. We propose that it has the potential to contribute to the decolonization agenda within Nigerian higher education. CCMAS offers a nationally mandated framework that explicitly seeks to ground medical training in locally relevant content and indigenous contexts, an acknowledgement, at the level of national regulatory policy, that Nigerian medical education requires fundamental reform. Its significance lies not only in its substantive provisions, but in what it represents institutionally: a formal opening through which decolonial principles can enter the curriculum with regulatory authority behind them (39).

However, we contend that CCMAS will require sustained monitoring, evaluation, and accountability mechanisms to move beyond policy aspiration toward genuine curricular transformation. How consistently CCMAS has been adopted across Nigeria's diverse medical schools, and whether it has produced measurable changes in curriculum content or pedagogical approach, remains an urgent question for future research.

A concrete evaluation agenda would assess CCMAS along at least four dimensions. First, adoption fidelity: whether medical schools have formally revised curricular documents, assessment blueprints, and course content to reflect CCMAS's locally relevant provisions verifiable through curriculum audits comparing pre- and post-CCMAS syllabi. Second, content change: the proportion of taught hours, case studies and assessment items that engage indigenous knowledge, local disease burden or Nigerian health-system context, trackable through structured curriculum mapping. Third, pedagogical change: whether teaching methods and faculty practice, and not only documented content, have shifted, assessable through classroom observation, student surveys, and faculty self-report. Fourth, downstream outcomes: graduate preparedness for Nigerian clinical contexts, measured through licensing-examination performance on locally relevant items, employer and preceptor evaluation, and, over a longer horizon, retention and practice-location data. The National Universities Commission, in collaboration with the Medical and Dental Council of Nigeria and individual institutions, is well placed to commission or mandate this evaluation, ideally through a standardized audit instrument applied across a representative sample of medical schools rather than relying on institutional self-report alone.

### 4.2 Indigenous language integration

The formal integration of indigenous languages into medical curricula is a high-priority and practically achievable decolonial intervention. Formalizing existing informal student practice through dedicated language training, multilingual clinical skills instruction, and explicit expectations for indigenous language use during placements would validate student behavior, improve clinical communication, and affirm the epistemic legitimacy of non-English expression within medical training (34). Studies by Thilakasiri et al. (40) extend this argument to simulation-based education, calling for multilingual instruction and locally contextualized scenarios that produce more authentic learning experiences. This is not a concession to linguistic diversity but a substantial decolonial intervention that transforms the epistemological assumptions underlying clinical education.

### 4.3 African philosophies and curriculum reorientation

African philosophical frameworks, most notably Ubuntu, encapsulating the relational principle that personhood and wellbeing are constituted through community and interdependence, offer a foundational reorientation for health professions education that goes beyond content diversification. Nel et al. (41) propose restructuring clinical training curricula around Ubuntu and related frameworks such as ujamaa (the philosophy of community/familyhood) and uhaki (the philosophy of social justice), centering communal wellbeing and relational ethics as core organizing principles rather than supplementary additions to a Western-derived curriculum. Razack et al. (42) similarly advocate for elevating locally situated African philosophical perspectives as legitimate anchors for equitable educational partnerships.

Community-based training programs, like the “Ife Philosophy” at the University of Ife in the 1970s, offered a complementary possibility, enhancing understanding of local health needs, promoting culturally responsive clinical reasoning, and connecting theoretical frameworks and the lived experiences of healthcare delivery in Nigerian communities (18, 43).

Within specialized fields such as emergency medicine and speech-language therapy, there are growing calls to embed indigenous knowledge systems and Afrocentric curricular modules into professional training (44, 45). These approaches share a common logic: that genuine decolonization requires not merely adding African contexts to existing frameworks but rebuilding those frameworks on African epistemological foundations.

### 4.4 Responsive global partnerships

International partnerships in medical education can either reinforce or disrupt colonial power relations depending on how they are structured. The Medical Education Partnership in Nigeria (MEPIN), a collaboration between the University of Ibadan and the Harvard School of Public Health, offers a Nigeria-specific example of this model, having been established to strengthen local medical education and research capacity rather than to impose externally designed curricula (46). Furthermore, evidence from Kenya indicates that the success of health professions training programs is closely linked to approaches that prioritize local knowledge systems and collaborative practice models over the imposition of Western frameworks (47). Reciprocal exchange programs intentionally designed around genuine power-sharing, in which international collaboration is structured around mutual learning rather than unidirectional knowledge transfer, offer a model for what decolonized global partnership in medical education might look like (48). Ebeye and Lee (30) argue that addressing brain drain's structural dimensions requires holding high-income countries accountable for strengthening the systems for which they recruit, positioning responsive global engagement as a decolonial political commitment rather than a technical matter of program design. Table 2 shows how each barrier identified maps onto a corresponding possibility.

**Table 2:** Proposed barriers-to-possibilities mapping framework.

Barrier	Corresponding possibility
1948 colonial curriculum legacy	CCMAS as institutional lever
Northern hegemony and epistemic asymmetry	African philosophies and curriculum reorientation
Brain drain as neo-colonialism	Responsive global partnership
English-medium gatekeeping	Regional indigenous language and/or Pidgin English integration
Institutional resistance and underfunding	Community-based training, faculty development, dedicated funding from Tertiary Education Trust Fund

### 4.5 Toward an operational framework for decolonization

To move from aspiration to accountability, we propose a three-tier framework that institutions and external stakeholders can use to characterize the depth of a given reform effort. Table 3 sets out these tiers and illustrative indicators for each, mapped against the institutional actions summarized in Figure 1.

**Table 3:** Three-tier framework for evaluating the depth of institutional reforms.

Tier	Definition	Illustrative indicator
Symbolic reform	Diverse content, guest lectures, or references to decolonization are added without altering assessment criteria, faculty practice, or institutional decision-making authority.	The reading list is diversified, but assessment blueprints and marking criteria are unchanged.
Substantive reform	Concrete curricular and structural changes are made—revised reading lists centering African scholars, indigenous knowledge holders formally integrated as instructors, locally developed teaching materials—without a corresponding shift in who holds authority over curricular or research agendas.	Indigenous knowledge holders co-teach a module, but curriculum design authority remains with externally accredited standards.
Transformative reform	Decision-making authority over curriculum design, assessment standards, funding allocation, and research agendas is redistributed towards Nigerian and African institutions and scholars, such that local priorities drive curricular design.	Local health priorities and Nigerian-led evaluation criteria, rather than international accreditation norms, determine what is taught and assessed.

This framework is intended as a practical self-assessment and accountability tool rather than a rigid classification: individual reforms may combine elements of more than one tier, and progress from symbolic towards transformative reform is likely to be incremental rather than immediate.

**Figure 1:**
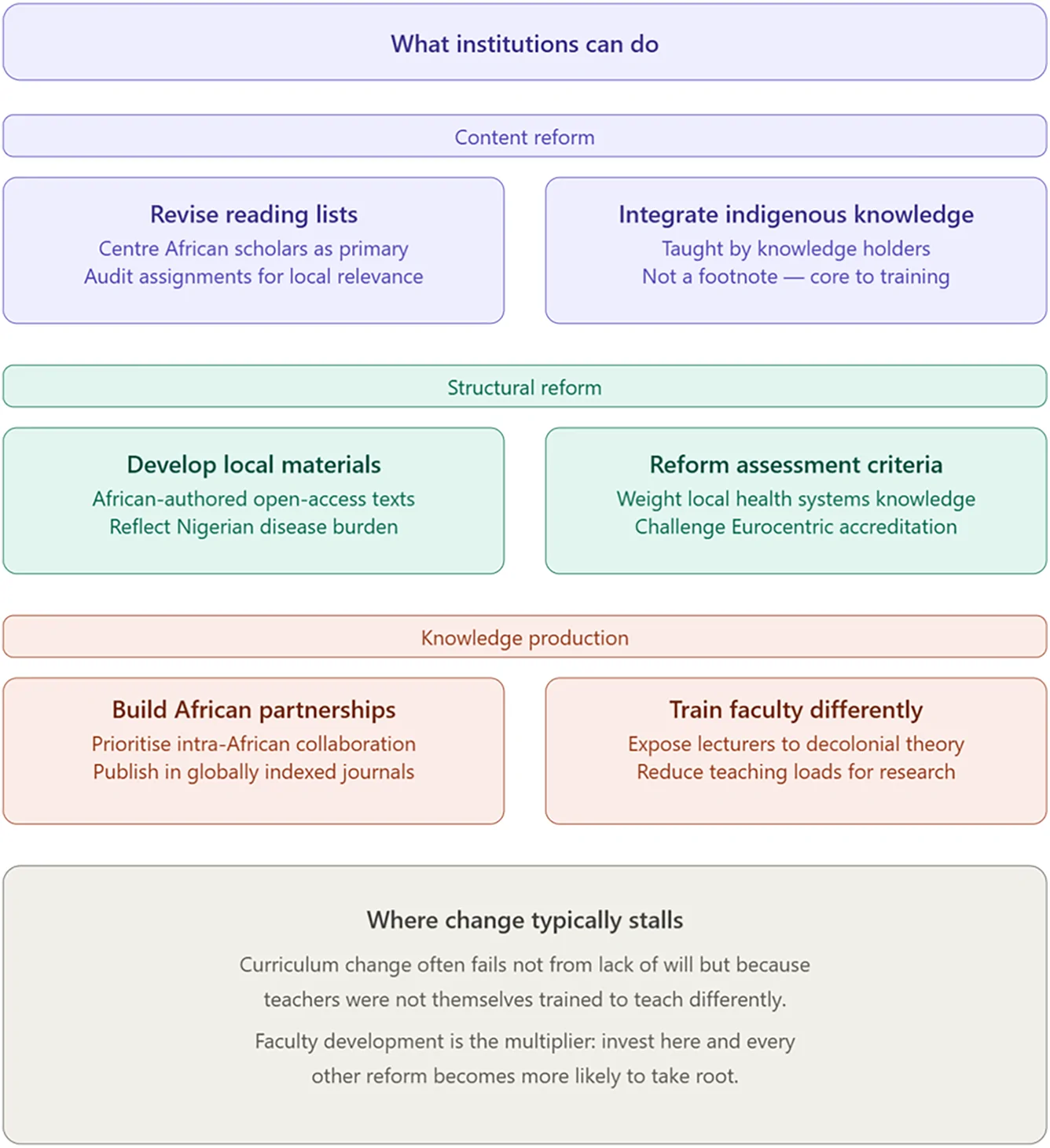
Actions institutions can take to decolonize Nigerian medical education. Actions are grouped under content reform, structural reform and knowledge production; curriculum reform typically stalls where faculty have not been trained to teach differently, making faculty development the multiplier investment.

## 5 Implications for research, policy, and practice

Advancing decolonization in Nigerian medical education requires coordinated action across research, policy, funding, and practice, rather than confinement to any single level. We argue that at the institutional level, individual educators can act now by incorporating locally relevant case studies, engaging indigenous knowledge holders in clinical teaching, and creating space for indigenous language use in supervised clinical placements without waiting for systemic change. Yet curriculum reform characteristically stalls not from lack of institutional will but because faculty themselves were not trained to teach differently. Faculty development in decolonial pedagogy is therefore the multiplier investment on which other reforms depend (14, 49). We propose that Tertiary Education Trust Fund resources support more decolonial work through additional dedicated funding.

Beyond the institutions, funders, policy makers, and researchers shape the wider conditions for reform. The specific actions available to each include: funders restructuring grant conditions and removing access barriers to African-led research; policymakers reforming accreditation and building regional coordination through bodies such as ECOWAS and the African Union; researchers centering lived experience and strengthening the evidence base while challenging the citation hierarchies that marginalize African scholarship (8, 29). What unites these actors is a shared risk: each can reproduce the very structures they intend to dismantle unless reform moves beyond consultation toward genuine Southern decision-making authority over funding, accreditation, and research agendas (50). Together, these actions represent an interconnected institutional commitment, one that requires sustained leadership, adequate resourcing, and the political will to prioritize local relevance. Figure 1 depicts an institutional framework for action on decolonizing medical education in Nigeria.

### 5.1 Limitations

This perspective is subject to limitations. First, as a narrative and interpretive analysis rather than a systematic or scoping review, the sources were selected purposively rather than through a comprehensive search strategy, and relevant Nigerian or African scholarship, particularly grey literature or work not indexed in English-language databases, may not have been captured. Second, several of the causal relationships proposed in this paper, for instance, between colonial curricular structures and clinical outcomes, patient trust, or graduate retention are plausible and consistent with the broader decolonization literature but are not established by direct Nigeria-specific outcome data; they should be read as hypotheses warranting empirical testing rather than as demonstrated effects. Third, the authors' positionality, while a source of grounded insight, does not encompass the perspectives of patients, indigenous knowledge holders, or students currently navigating the system; we have sought to represent these voices through the literature engaged with here, but this is not a substitute for direct engagement with those groups. Finally, the comparative material drawn from Kenya, South Africa, Zambia, and North Africa is illustrative rather than a systematic cross-national comparison and dedicated comparative research would be needed to substantiate the parallels drawn.

## 6 Conclusion

Nigerian medical education remains shaped by a colonial curriculum legacy that has persisted largely intact for over seven decades. The barriers to decolonization are deep, systemic, and mutually reinforcing, rooted in epistemological asymmetry, institutional conservatism, structural under-resourcing, and the neo-colonial dynamics of global knowledge production and physician migration. These are not problems that curriculum diversification alone can address. They require a fundamental reimagining of what Nigerian medical education is for, whose knowledge it draws on, and whose health needs it is designed to serve.

The possibilities of transformation are real and growing. CCMAS, indigenous language integration, African philosophical frameworks, community-based training, and reciprocal global partnerships all represent genuine entry points for decolonial change within Nigerian medical education. But possibilities become transformation only when they are implemented with institutional commitment, adequately resourced, rigorously evaluated, and led by Nigerian and African voices with the authority and expertise to define what decolonized medical education means in this context.

Nigeria's scale, its colonial history, distinctive health burden, and the growing body of Nigerian and African scholarly voices engaging with decolonial theory make it both an urgent and a generative site for this work. The evidence base that would support and guide transformation remains underdeveloped. This perspective has sought to name the argument clearly and to make visible what remains to be done, with the conviction that clarity about the problem is a necessary first step towards its resolution.

## Data Availability

No datasets were generated or analyzed for this article. Further inquiries can be directed to the corresponding author.
